# A dangerous liaison: Leptin and sPLA2-IIA join forces to induce proliferation and migration of astrocytoma cells

**DOI:** 10.1371/journal.pone.0170675

**Published:** 2017-03-01

**Authors:** Rubén Martín, Claudia Cordova, Beatriz Gutiérrez, Marita Hernández, María L. Nieto

**Affiliations:** Instituto de Biología y Genética Molecular (IBGM), CSIC-UVa, Valladolid, Spain; Chang Gung University of Science and Technology, TAIWAN

## Abstract

Glioblastoma, the most aggressive type of primary brain tumour, shows worse prognosis linked to diabetes or obesity persistence. These pathologies are chronic inflammatory conditions characterized by altered profiles of inflammatory mediators, including leptin and secreted phospholipase A_2_-IIA (sPLA_2_-IIA). Both proteins, in turn, display diverse pro-cancer properties in different cell types, including astrocytes. Herein, to understand the underlying relationship between obesity and brain tumors, we investigated the effect of leptin, alone or in combination with sPLA_2_-IIA on astrocytoma cell functions. sPLA_2_-IIA induced up-regulation of leptin receptors in 1321N1 human astrocytoma cells. Leptin, as well as sPLA_2_-IIA, increased growth and migration in these cells, through activation/phosphorylation of key proteins of survival cascades. Leptin, at concentrations with minimal or no activating effects on astrocytoma cells, enhanced growth and migration promoted by low doses of sPLA_2_-IIA. sPLA_2_-IIA alone induced a transient phosphorylation pattern in the Src/ERK/Akt/mTOR/p70S6K/rS6 pathway through EGFR transactivation, and co-addition of leptin resulted in a sustained phosphorylation of these signaling regulators. Mechanistically, EGFR transactivation and tyrosine- and serine/threonine-protein phosphatases revealed a key role in this leptin-sPLA_2_-IIA cross-talk. This cooperative partnership between both proteins was also found in primary astrocytes. These findings thus indicate that the adipokine leptin, by increasing the susceptibility of cells to inflammatory mediators, could contribute to worsen the prognosis of tumoral and neurodegenerative processes, being a potential mediator of some obesity-related medical complications.

## Introduction

Over the last years, many studies have stated a harmful synergy among cancer, obesity and diabetes: individuals with diabetes and elevated body mass index are more likely to develop cancer; and cancer patients, who also suffer from diabetes or obesity, show a higher risk of mortality than non-diabetic and non-obese ones [1–3]. According to this, a recent study has demonstrated that in high grade glioma patients, pre-existing diabetes and obesity are independent risk factors for early progression and death [4]. Glioblastoma is the most common primary adult brain cancer with an extremely poor prognosis. Although it rarely metastasizes, it spreads aggressively within the brain, so it can rarely be totally removed using surgery. For this reason, understanding the mechanisms underlying this prognosis is a major challenge in order to find new strategies to control the neoplastic process.

Obesity is a systemic low-grade inflammatory disease characterised by sustained levels of circulating inflammatory proteins [5]. This results in a pro-tumorigenic environment which can play a role in malignant transformation and/or cancer progression. Among these active biological molecules, leptin and secreted phospholipase A_2_-IIA (sPLA_2_-IIA, sPLA_2_) have been found elevated in obese individuals and some forms of cancer [6–11].

Leptin plays an important role in the regulation of body weight homeostasis [12]. Classically produced by adipose tissue, leptin is released into the circulation to act both peripherally and in the brain [13]. However, finding leptin in blood leaving the brain suggests that leptin may also be synthesized by brain tissues [14]. In fact, in healthy individuals leptin released by the brain makes up/constitutes more that 40% of the whole plasma leptin, being this contribution remarkably higher in obese than in non-obese males ref [15]. Beside its link to obesity, leptin may also play a crucial role in cancer initiation, progression or in metastatic development. The biological function of leptin is triggered through its cell surface receptors (ObR) [16]. It has been described that the leptin/ObR system is over-expressed in brain cancer and its expression correlates with the degree of malignancy [10]. Leptin can amplify some oncogenic pathways via transactivation of receptors, and it may even cooperate with cytokines to amplify the inflammatory response [17–19].

sPLA_2_-IIA is an acute phase reactant found increased in numerous inflammatory conditions. Many studies suggest its involvement in carcinogenesis, although its specific role mediating pro- or antitumoral signaling, depends on the type of cancer [8,9]. Its expression level has been related to disease prognosis, and in some tumor types it is also considered a marker of metastasis [20–22]. sPLA_2_-IIA leads to excessive proliferation and survival signals in tumoral cells including astrocytomas [23].

Interestingly, individual effects of leptin and sPLA_2_-IIA have been studied in different established cell lines in vitro. However, the precise interaction among them and how their signaling cross-talk influences cell growth and migration is poorly understood, even though, in pathological conditions, it is their interplay that may activate intracellular pathways converging to promote tumor progression and metastasis.

This study characterizes the link between leptin and sPLA_2_-IIA on 1321N1 astrocytoma cells. Leptin not only increases cell growth, migration and activation of the classical proliferation cascades, but it also enhances biological responses of sPLA_2_-IIA by prolonging the temporal pattern of EGFR, ERK, Akt/mTOR and p70S6K/rS6 activation. This is the first evidence to demonstrate the relationship between two proteins present in the tumor microenvironment, leptin and sPLA_2_-IIA, and the effect of this tandem on tumor progression.

## Materials and methods

### Materials

A C127 mouse fibroblast cell line stably transfected with the coding sequence of sPLA_2_-IIA from human placenta was kindly provided by Dr. Jean-Luc Olivier and used as a source of the human recombinant enzyme, which was obtained and purified as described previously [24].

Rapamycin, leptin, ECL detection system, Hybond-P membrane and other chemicals were from Sigma Chemical Co. PD98059 and AG1478 and AG1296 inhibitors were from Tocris Bioscience. DMEM and the cell culture supplements, including foetal calf serum (FCS) were from Lonza.

### Cell culture

1321N1 human astrocytoma cells, a gift from Dr. JH Brown (UCSD, San Diego, CA, USA), were cultured in DMEM supplemented with 100 U/ml penicillin/streptomycin, and 10% heat-inactivated fetal calf serum (FCS) at 37°C in 5% CO_2_. Primary mouse astrocytes were obtained as described previously [25]. More than 95% of cells were stained for astrocyte specific glial fibrillary acidic protein (Santa Cruz Biotechnology Inc).

Primary astrocytes and astrocytoma cells were incubated in serum-free medium 24 h before the experiments, and then stimulated as indicated, in the presence or absence of inhibitors.

### Western blot analysis

Cells were lysed in Laemmli buffer and subjected to sPLA_2_-IIA SDS-polyacrylamide gel electrophoresis. Western blot analysis was performed with the following antibodies: phospho-Akt (Ser473), Akt, phospho-ERK1/2 (Thr202/Tyr204), phospho-P70S6K (Thr389), phospho-rS6 (Ser235/236) (Cell Signaling Technology, Inc), ERK (Zymed Laboratories), and actin (Santa Cruz Biotechnology Inc). Protein levels were detected with HRP-conjugated secondary antibodies, and visualized using the ECL detection system according to the manufacturer's instructions.

### Flow cytometric analysis

Serum-starved cells were incubated in medium with EGF (0.4 μM), sPLA_2_-IIA (0,5 μg/ml), PDGF (1 μM) or leptin (0.5 μM), for different periods of time at 37°C. Cells to analyze the expression of leptin receptor were fixed with 4% paraformaldehyde and 0.2% Triton X-100 in PBS for 15 min at room temperature, before incubation with anti-mouse ObR antibody (R&D Systems) for 1 h at 4°C. For EGFR and Src phosphorylation analysis, cells were fixed in 4% paraformaldehyde for 15 min, washed with PBS, permeabilized with 0.3% Triton X-100 for 5 min, washed, incubated with anti-phospho EGFR (Tyr1173), EGFR (Tyr845), EGFR (Tyr1068) or Src (Tyr416) antibody (Santa Cruz Biotechnology Inc) for 1 h at 4°C, and then with a fluorescein isothiocyanate (FITC)-labelled secondary antibody (Sigma) for 45 min at 4°C. After washing, cells were analyzed with a Flow Cytometer (Gallios^TM^; Beckman Coulter). Data analysis was performed using WinMDI 2.7 software.

### Immunofluorescence studies

Serum-starved 1321N1 cells, on 18-mm cover glass coverslips, were fixed in cold 4% paraformaldehyde for 10 min, permeabilized in 0.3% Triton X-100 for 10 min and blocked with 3% BSA for 1h. Immunostaining with ObR antibody (H-300; Santa Cruz Biotechnology Inc) was performed at room temperature for 30 min, followed by incubation with FITC-conjugated secondary antibody. Cells were then analyzed under a Nikon Eclipse 90i (Nikon Instruments, Inc.).

### Proliferation assay

Cell proliferation was quantified using the Promega kit, Cell Titer 96^R^Aqueous One Solution Cell Proliferation Assay, according to the manufacturer's recommendations. Briefly, serum-starved 1321N1 astrocytoma cells, seeded in 96-well tissue culture plates (5×10^4^ cells/ml), were treated in triplicate with sPLA_2_-IIA, EGF, leptin or combinations, in the presence or absence of inhibitors. After 24h of incubation, formazan product formation was assayed by recording the absorbance at 490 nm in a 96-well plate reader, as an assessment of the number of metabolically active cells.

### Chemotaxis assay

Cell migration assays were performed using 24-well Transwell plates with 8μm-pore polycarbonate membrane filters, following the manufacturer’s instructions. Lower wells were filled with medium containing vehicle, 50% FCS or leptin, as indicated. Cells (1x10^6^ cells/ml) were added to the upper chamber. Before the migration assay was performed, and when indicated, cells were pretreated for 30 min with selective inhibitors, and then stimulated with sPLA_2_-IIA or leptin for 30 additional min. Migration was allowed for 4 h at 37°C in 5% CO_2_. After incubation, nonmigrating cells were scraped off from the upper surface of the filter. Migrating cells were fixed with 4% paraformaldehyde and stained with 0.1% Crystal Violet in 20% methanol-PBS for 3 min. To quantify migration, the bound stain was solubilized and absorbance measured at 590 nm. Each experiment was repeated at least three times in triplicate. Chemotactic index was calculated from the number of cells migrating towards chemoattractant/number of cells migrating towards vehicle control.

### Phosphatase assay

Cells were resuspended with PBS, subjected to three rounds of freeze/thaw and then sonicated for 10s. Serine/threonine and tyrosine phosphatase activities were measured using a commercially available specific phosphatase assay kits (Molecular Probes Inc). Five micrograms of protein were used in the assay. The generated fluorescent product was determined using a fluorometer (Ex 358 nm and Em 452 nm).

### Data presentation

The results are presented as the mean ± SD or as representative results from more than three different batches of cells, unless otherwise stated. Statistical significance was assessed by using ANOVA followed by Bonferroni post-hoc testing, with Graph Pad Prism software, version 5 (GraphPad Software Inc).

## Results

### Leptin stimulates EGFR-independent proliferation of 1321N1 cells

**Fig 1:**
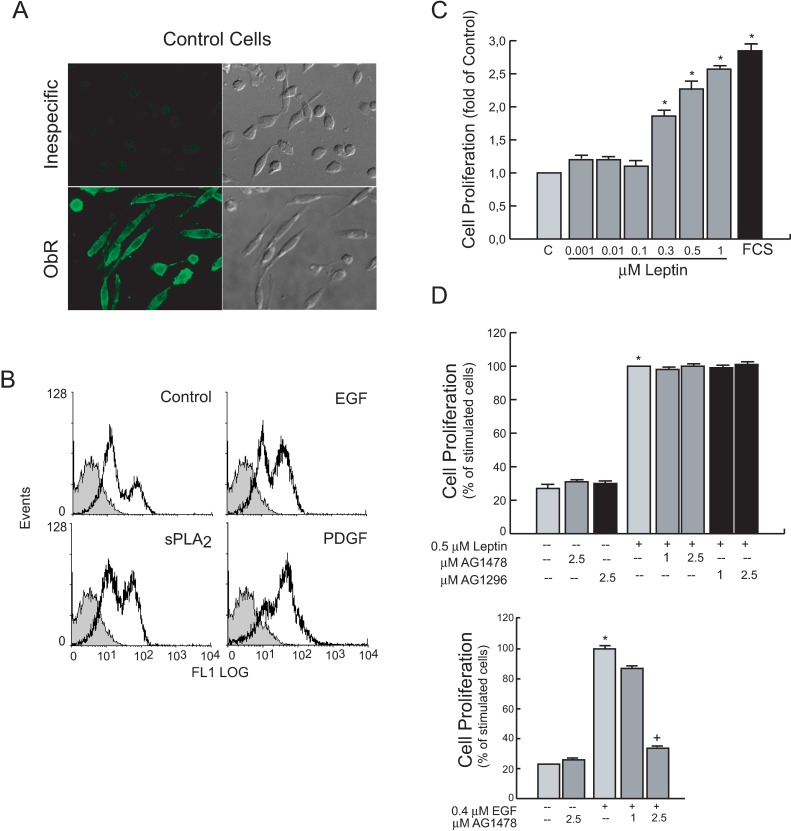
1321N1 astrocytoma cells express the leptin receptor (ObR) and leptin stimulates EGFR-independent cell proliferation. (A) 1321N1 cells immunofluorescence for ObR expression without and with primary anti-ObR antibody (H-300). Representative histograms. Unspecific staining, solid grey curves. OB-R expression in cells treated as mentioned in the plot, open black curves (B) Cells stimulated for 18 h with the indicated agonist: Expression of ObR was analyzed by flow cytometry. Serum-starved 1321N1 cells were stimulated for 24 h with increasing concentrations of leptin (C), or with the indicated agonist in the presence or absence of specific inhibitors (D). Cell proliferation was measured as explained in Materials and Methods. *p≤0.001 vs untreated control, ^+^p≤0.001 vs EGF, n = 3

We first checked for the constitutive expression of the leptin receptor (ObR) on human 1321N1 astrocytoma cells (Fig 1A). Then, we examined whether this expression could be altered during exposure to proliferative agents also present in the tumor microenvironment. As shown in Fig 1B, we found that cell stimulation increased constitutive ObR expression levels on the cell surface. After a 24 h-stimulation with sPLA_2_-IIA, EGF or PGDR the population of cells expressing high levels of OB-R increased from the 18% observed in control, to 42%, 45% and 66%, respectively.

We next analyzed leptin-induced proliferative responses in these cells, since cell growth is a well-known effect of this adipokine in tumoral cells. As shown in Fig 1C, leptin enhanced 1321N1 cell proliferation in a dose-dependent manner and reached a 2.1 ± 0.4-fold (p≤0.001) increase when stimulated with 0.5 μM leptin for 24h.

Moreover, we analyzed whether leptin-induced proliferative responses in 1321N1 cells involved EGFR or PDGFR, important signaling mechanisms in cancer cells. We incubated cells for 30 min in the presence of different doses of the EGFR kinase inhibitor AG1478 or the PDGFR kinase inhibitor AG1296, before stimulation with 0.5 μM leptin or 0.4 μM EGF for 24h. The presence of specific inhibitors did not affect proliferation induced by leptin, whereas, as expected, pretreatment with the EGFR kinase inhibitor AG1478 inhibited the EGF-induced signalling and proliferation (Fig 1D).

### Leptin–induced Proliferation of 1321N1 cells requires the activity of classical ERK, Akt, pathways

**Fig 2:**
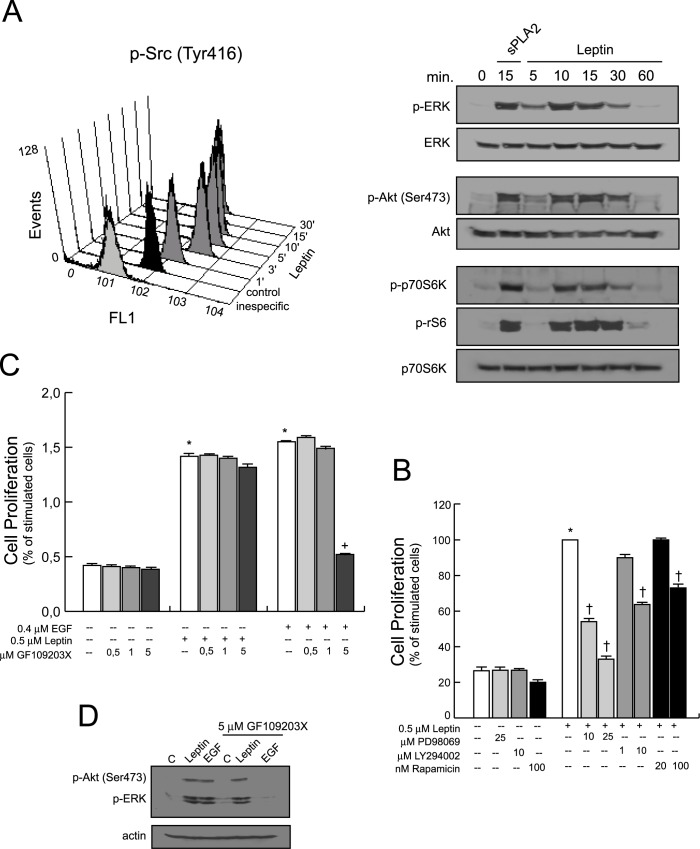
Intracellular pathways involved in the growth promoting effect of leptin in 1321N1 cells. (A) Cells were stimulated with 0.5 μM of leptin for indicated times intervals, Src phosphorylation was analyzed by flow cytometry analysis, and ERK, Akt, P70S6K and rS6 phosphorylation were determined by immunoblotting, using phospho-specific antibodies. (B) Cells were treated with the inhibitors, PD98059, LY294002 or rapamycin before treatment with leptin, or with (C) GF109203X before treatment with leptin or EGF. After 24 h, cell proliferation was measured as explained in Materials and Methods. (D) Cells treated as in (C) for 15 min. Protein phosphorylation was determined by immunoblotting, using phospho-specific antibodies. *p≤0.001 vs untreated control, ^+^p≤0.001 vs EGF, ^†^p≤0.001 vs leptin treated, n = 3

Next, to investigate whether the activation of Src/ERK/Akt/mTOR kinases were key elements of the leptin induced proliferation in 1321N1 astrocytoma cells, they were incubated in the absence or presence of an optimal concentration of human recombinant leptin (0.5 μM) at different times. Flow cytometry analysis revealed that resting 1321N1 cells constitutively expressed Tyr 416-phophorylated Src. Levels increased 4-fold over control by 3 min after leptin treatment, and declined to near baseline levels by 30 min (Fig 2A).

We also found that leptin treatment immediately increased the presence of phosphorylated extracellular signal regulated kinases (ERK, p-ERK), reaching a maximum level of phosphorylation around 10 min, decreasing afterwards to reach basal levels, approximately at 60 min (Fig 2A, S1A Fig).

Similarly, we observed Akt phosphorylation on Ser473 with a time course that paralleled ERK activation/phosphorylation profile. In addition, the rapamycin-sensitive molecules P70 ribosomal protein S6 kinase (P70S6K) and S6 ribosomal protein (rS6) were also phosphorylated upon leptin exposure following a similar pattern, with a maximal effect at 15 min, which almost disappeared 60 min after incubation. 1321N1 cells stimulated with 0.5 μg/ml of sPLA_2_-IIA for 15 min were used as a positive control.

To identify the role of these signalling elements on leptin-induced astrocytoma cell growth, the mitogenic response of 1321N1 cells to 0.5 μM of leptin was tested in the presence or absence of several selective pharmacological inhibitors. As shown in Fig 2B, leptin-induced proliferation was almost abolished by inhibition of ERK with the MEK inhibitor PD98059. Inhibition of the PI3-kinase/Akt pathway with LY 294002, or with the specific mTOR inhibitor rapamycin, also diminished cell proliferation in a concentration-dependent manner (Fig 2B). We also found that 20 μM of the Src inhibitor, PP2, blocked leptin-induced cell proliferation (not shown).

However, the presence of different doses of the PKC inhibitor GF109203X did not markedly affect the response induced by leptin in 1321N1 cells (p>0.05) (Fig 2C and 2D, S1B Fig), while EGF induced cell proliferation, as well as ERK and Akt phosphorylation, was abrogated by pre-treatment of the cells with 5 μM of GF109203X (**p≤0.001).

**Fig 3:**
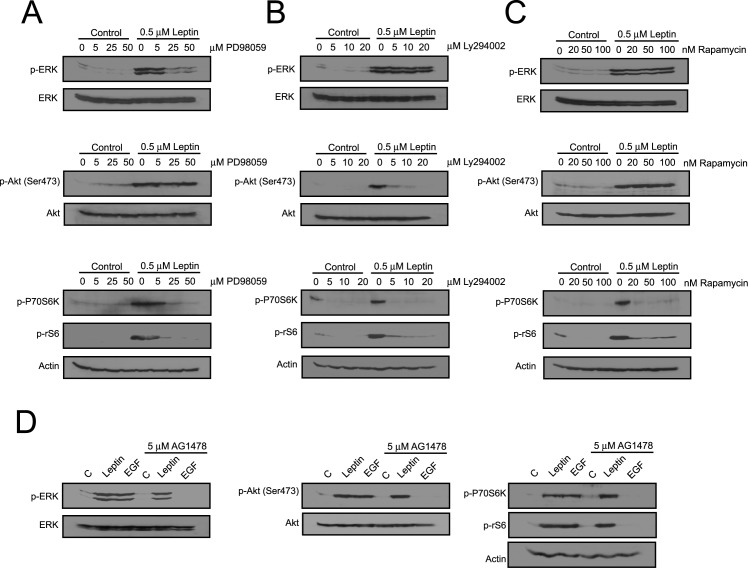
Effect of kinase inhibitors on the intracellular signaling mediators activated in leptin-treated cells. 1321N1 cells were incubated without or with 0.5 μM of leptin or 0.4 μM of EGF for 15 min in the presence or absence of the indicated inhibitors. Lysates were analysed by western blotting with the specified antibodies.

To understand and further characterize the activation sequence of leptin in 1321N1 cells, we incubated them in the presence or absence of selective inhibitors. We observed that ERK phosphorylation was abolished by pretreatment of the cells with the MEK inhibitor PD98059, which also abrogated activation of p70S6K and rS6, leaving Akt phosphorylation unaffected (Fig 3A, S2A Fig). We also found that PP2, the Src inhibitor, blocked ERK phosphorylation (data not shown). LY294002, the PI3K inhibitor, significantly reduced the phosphorylation of Akt/p70S6K/rS6 signaling proteins, but it did not affect ERK (Fig 3B, S2B Fig) or Src (data not shown) phosphorylation induced by leptin. In addition, rapamycin, the mTOR inhibitor, also reduced p70S6K and rS6 phosphorylation (Fig 3C, S2C Fig).

Finally and as expected, the presence of the EGFR inhibitor, AG1478, did not affect leptin-stimulated phosphorylation/activation of these signaling molecules, while it abolished EGF-stimulated phosphorylation (Fig 3D, S2D Fig). The PDGFR kinase inhibitor AG1296 did not abolish the immediate phosphorylation of the studied kinases either (not shown).

### Leptin synergizes with sPLA_2_-IIA to induce proliferation of 1321N1 cells

**Fig 4:**
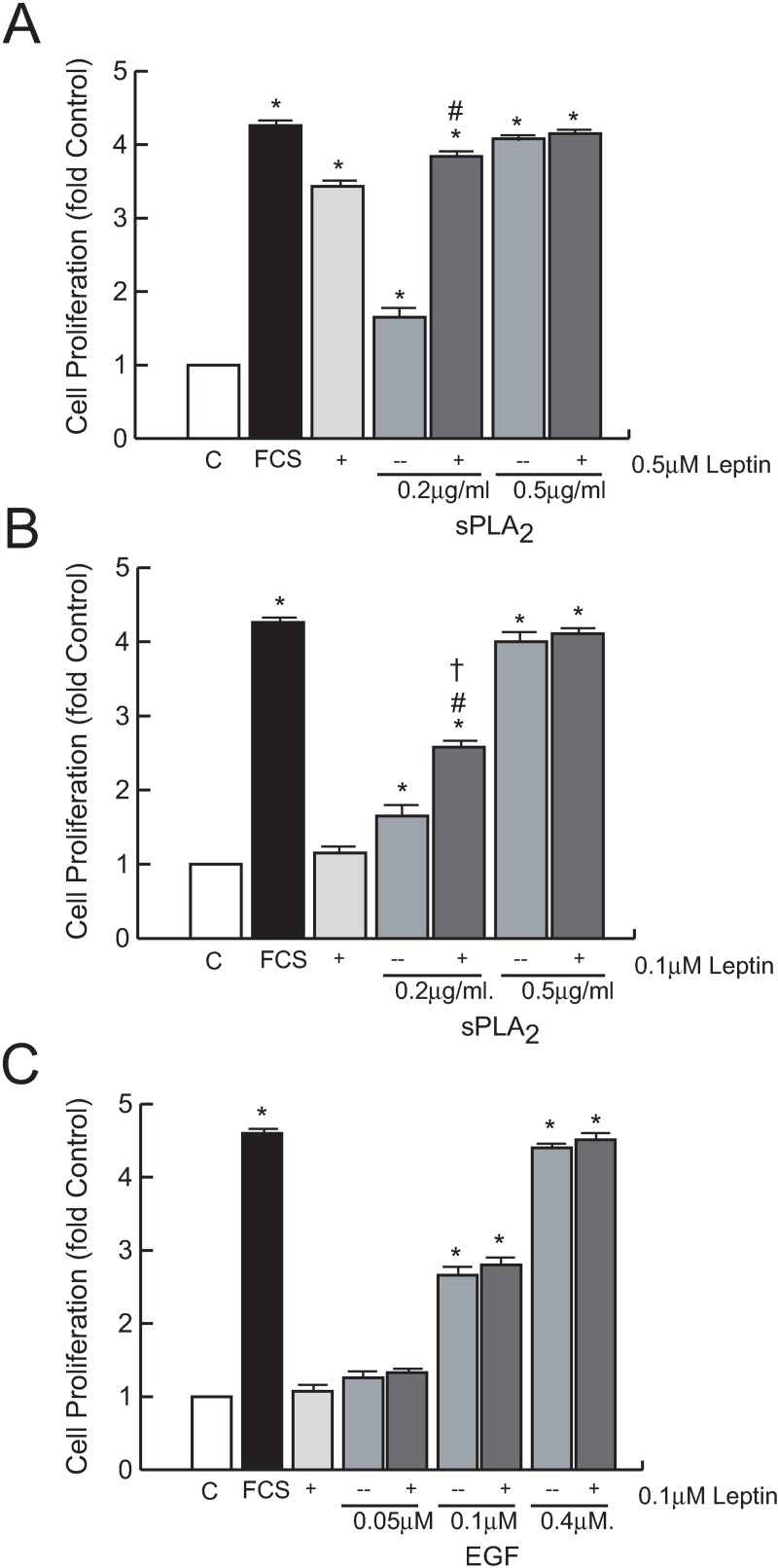
Effect of leptin in sPLA_2_-IIA- and EGF-induced cell proliferation. Serum-starved 1321N1 cells were stimulated for 24 h with the indicated concentrations of leptin, sPLA_2_-IIA (sPLA_2_) and EGF. Cell proliferation was measured as explained in Materials and Methods. *p≤0.001 vs untreated control, ^#^p≤0.001 vs sPLA_2_ alone, ^†^p≤0.001 vs leptin alone, n = 3

Next, we compared the proliferative activity of leptin in 1321N1 cells with known mitogens implicated in tumor pathogenesis such as EGF or sPLA_2_-IIA (Fig 4). As demonstrated previously, 0.5 μg/ml of sPLA_2_-IIA induced a strong mitogenic response in a serum-free cell culture [23,24]. The simultaneous presence of 0.5 μM leptin and sPLA_2_-IIA, either at optimal (0.5 μg/ml) or suboptimal (0.2 μg/ml) concentrations did not yield any significant additive effect (Fig 4A). Interestingly, although the addition of a suboptimal dose of leptin, 0.1 μM, to the optimal concentration of sPLA_2_-IIA (0.5 μg/ml) had no major effect on the proliferative response of the sPLA_2_-IIA, when it was combined with the lowest dose of the phospholipase, 0.2 μg/ml, the proliferative response induced was significantly higher than promoted by either agonist alone (Fig 4B), suggesting the possibility that leptin synergizes with sPLA_2_-IIA to stimulate growth.

Although EGF also induced a dose-dependent mitogenic response in the serum-free culture, the addition of 0.1 μM of leptin had no further effect on the proliferative activity of EGF at any dose tested (Fig 4C), showing a specific link between sPLA_2_ and leptin signaling.

### Leptin modulates migration of untreated and sPLA_2_-II-treated 1321N1 cells

We focussed on leptin and sPLA_2_-IIA, alone or combined, in the search for factors and mechanisms involved in the invasive behaviour of brain tumors.

**Fig 5:**
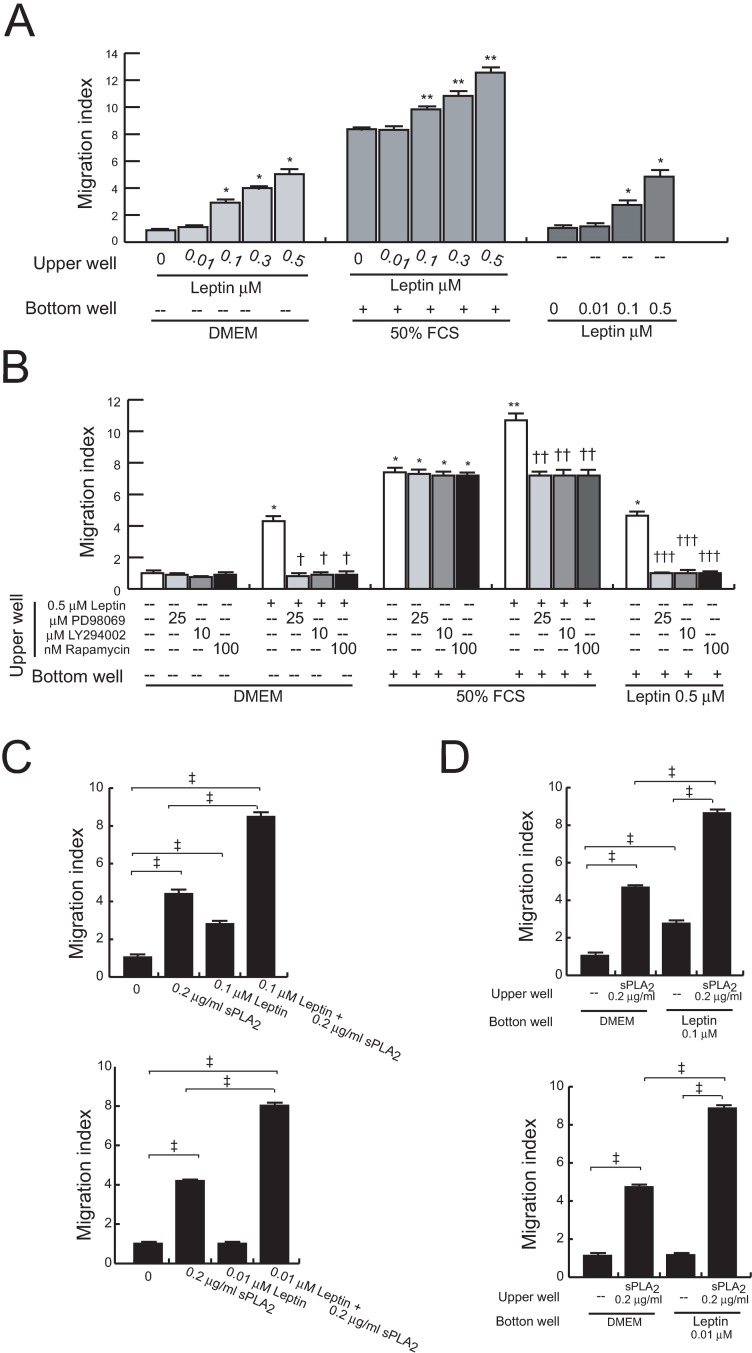
Effects of leptin on the migration of untreated and sPLA_2_-IIA-treated cells. Cell mobility was assayed using the 96-well Boyden chamber system. Serum-starved 1321N1 cells were untreated or treated with the indicated concentration of leptin for 30 min, in the absence (A) or presence of kinase inhibitors (B). Cells were allowed to migrate for 2 h to DMEM, FCS or leptin. *p<0.001 vs untreated cell towards DMEM, **p<0.001 vs untreated cells towards FCS, ^†^p<0.001 vs leptin-treated cell towards DMEM, ^††^p<0.001 vs leptin-treated cell towards FCS, ^†††^p≤0.001 vs untreated cell towards leptin, n = 3. Serum-starved 1321N1 cells were treated as indicated for 30 min (loaded into top wells), and then allowed to migrate for 2 h to DMEM (C), or treated with medium or sPLA_2_-IIA (sPLA_2_) for 30 min (loaded into top wells), and then allowed to migrate for 2 h to DMEM or leptin (loaded into the bottom wells) (D). ^‡^ p≤0.001, n = 3.

Experiments using leptin as a chemoattractant revealed a dose-dependent chemotactic motility on these cells (Fig 5A). Besides, we also found that leptin increases their spontaneous and FCS-mediated migratory capabilities in a concentration dependent manner. Pretreatment of 1321N1 cells with the selective pharmacological inhibitors LY294002, PD98059 or rapamycin reduced the chemoattractant capacity of leptin, as well as leptin-induced migration, both baseline and FCS-mediated (Fig 5B).

Before searching for a cooperative effect between leptin and sPLA_2_-IIA, we assessed the capacity of sPLA_2_-IIA to modulate 1321N1 migration. As shown in Table 1, sPLA_2_-IIA pretreatment stimulated both spontaneous and FCS-induced cell migration. Then, cells were stimulated with a combination of sPLA_2_-IIA and leptin, at concentrations eliciting none or minimal migratory responses, and we observed an increased cell mobilization compared to either stimulus alone (Fig 5C). Likewise, addition of leptin to the lower transwell chamber, at doses eliciting minor or no detectable chemoattractant effect in untreated cells, further increased the migration of sPLA_2_-IIA-treated 1321N1 cells (Fig 5D).

**Table 1:** sPLA_2_ treatment promotes 1321N1 cell migration.

Cell treatment	Migration Index	
	DMEM	DMEM + 50% FCS
None	1 ± 0.01	8.48 ± 0.02^*^
0.2μg/ml sPLA_2_	4.46 ± 0.01^*^	8.48 ± 0.02^*^
1μg/ml sPLA_2_	8.86 ± 0.03^*^	12.86 ± 0.02^*^^,^^**^

Cells were treated with different doses of sPLA_2_-IIA (sPLA_2_) for 30 min at 37°C, and then allowed to migrate towards DMEM or DMEM+FCS for 2h at 37°C (see “Experimental Procedures” for calculation of relative migratory index). The data represent the mean ± S.D for four independent experiments.

* and ** indicates p<0.001 relative to untreated cells migrating towards DMEM or DMEM+FCS, respectively.

### Leptin modifies the temporal pattern of key signal-transduction elements activated by sPLA_2_-IIA on 1321N1 cells

**Fig 6:**
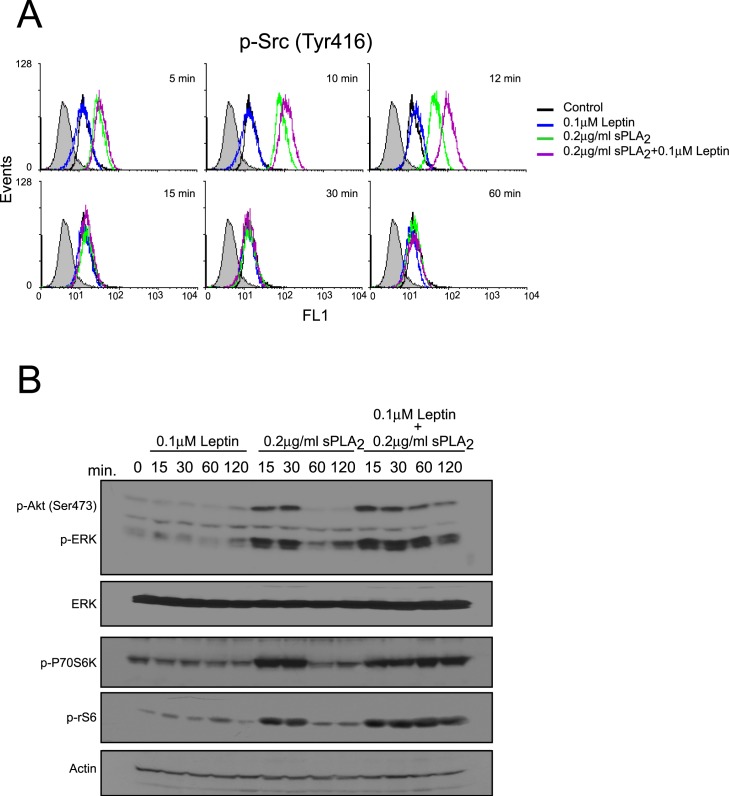
Effect of leptin+sPLA_2_-IIA treatment on the activation of intracellular kinases in 1321N1 cells. 1321N1 cells were stimulated with 0.1 μM of leptin, 0.2 μg/ml of sPLA_2_-IIA (sPLA_2_) or both, for the indicated time intervals. Src phosphorylation was analyzed by flow cytometry analysis (A), and ERK, Akt, P70S6K and rS6 phosphorylation were determined by immunoblotting, using phospho-specific antibodies (B).

To elucidate the intracellular signaling mechanisms involved in the ability of leptin to potentiate sPLA_2_-IIA-induced biological responses, we investigated whether 0.1 μM leptin altered the time course of Src, ERK, Akt or p70S6 kinase activation induced by 0.2 μg/ml sPLA_2_-IIA. As shown in Fig 6 and S3 Fig, there was little or no increase in the magnitude of phosphorylation of the kinases studied after addition of a suboptimal dose of leptin (0.1 μM) to 1321N1 astrocytoma cells; however 0.2 μg/ml sPLA_2_-IIA led to a rapid transient activation of all the above mentioned kinases. Interestingly, incubation of the cells with leptin and sPLA_2_-IIA simultaneously led to an immediate and sustained increase in the phosphorylation of ERK, Akt and p70S6 kinase, up to 2h post stimulation. The effect on Src phosphorylation under these conditions was less pronounced.

These results may suggest that the ability of leptin to potentiate sPLA_2_-IIA-induced proliferation is due to the prolongation of sPLA_2_-IIA-induced kinases phosphorylation/activation.

Having previously established that sPLA_2_-IIA acts as a potent modulator of cell proliferation by inducing EGFR transactivation, we proposed to identify the potential role of EGFR in mediating the cooperative actions of leptin and sPLA_2_-IIA.

**Fig 7:**
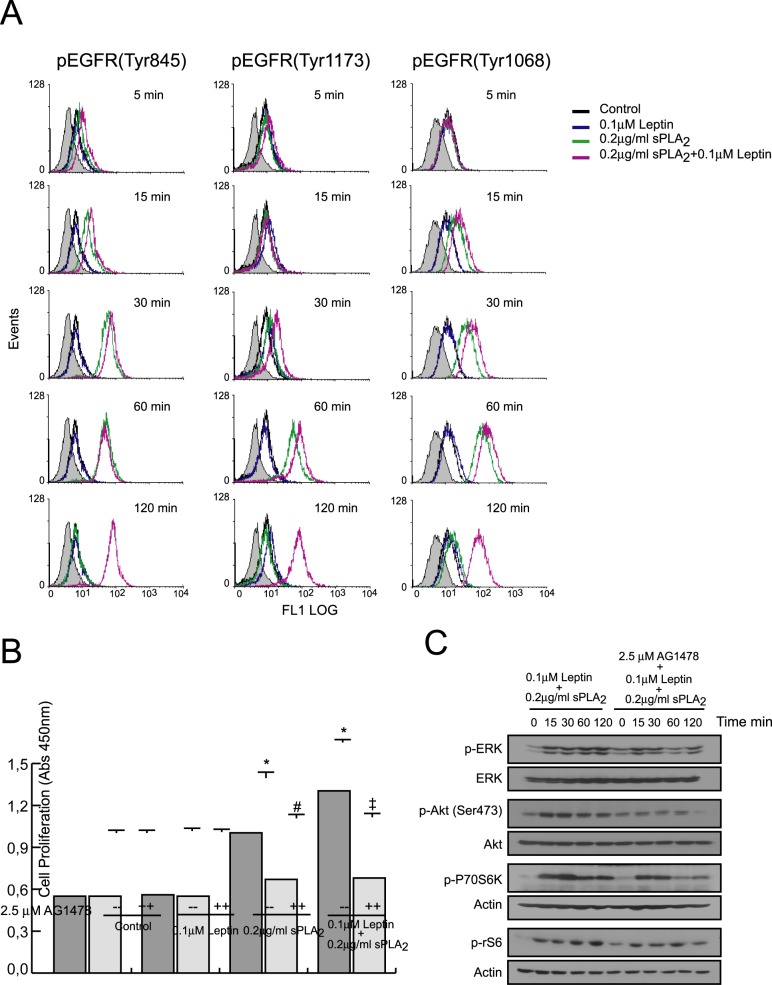
Effect of leptin+sPLA_2_-IIA treatment on EGFR transactivation in 1321N1 cells. 1321N1 cells were stimulated with 0.1 μM of leptin, 0.2 μg/ml of sPLA_2_-IIA (sPLA_2_) or both, in the presence or absence of 2.5 μM of AG1478. (A) At the indicated times, EGFR phosphorylation was analyzed by flow cytometry analysis. (B) After 24 h cell proliferation was measured as explained in Materials and Methods. *p≤0.001 vs control without inhibitor, ^#^p≤0.001 vs sPLA_2_ treated without inhibitor and ^‡^p≤0.001 vs leptin+sPLA_2_ treated without inhibitor. (C) Phosphorylation of signaling proteins was determined by immunoblotting at the indicated times.

A significant and sustained increase in EGFR phosphorylation (Tyr 1176, Tyr 1068 and Tyr 845) was observed upon sPLA_2_-IIA stimulation, either alone or in combination with leptin, while leptin alone, as expected, showed no effect (Fig 7A). However, whereas in sPLA_2_-IIA-stimulated cells EGFR phosphorylation level declined after 60 min of treatment, in cells treated with sPLA_2_-IIA+leptin the tyrosine phosphorylation status of EGFR was maintained, at least, 2h after stimulation.

In keeping with these results, we showed that sPLA_2_-IIA- and sPLA_2_-IIA+leptin-mediated responses were sensitive to EGFR inhibition (Fig 7B and 7C, S4 Fig). We found that AG1478 was able to inhibit not only sPLA_2_-IIA-stimulated growth, but also the synergistic effect of leptin and sPLA_2_-IIA on 1321N1 cell proliferation. In addition, the persistent activation of the selected signaling proteins induced by the simultaneous addition of sPLA_2_-IIA and leptin was also dramatically reduced, particularly at later time points, where their phosphorylation was close to basal levels.

### Effect of leptin on protein phosphatase activity

**Fig 8:**
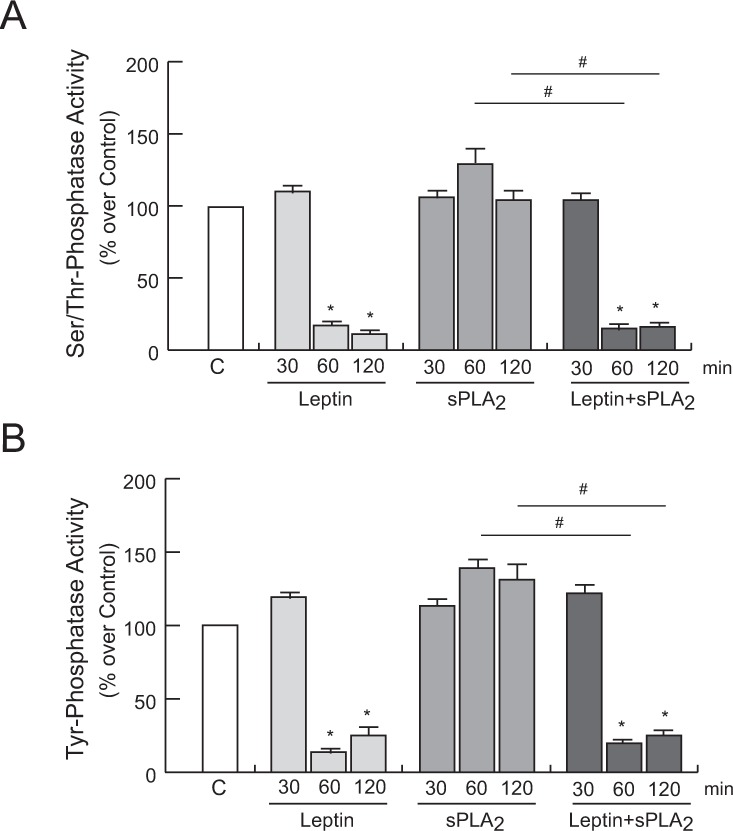
Effect of leptin and sPLA_2_-IIA on phosphatases activity in 1321N1 cells. 1321N1 cells were stimulated with 0.1 μM of leptin, 0.2 μg/ml of sPLA_2_-IIA (sPLA_2_) or both, for the indicated times. Ser/Thr phosphatase (A) and Tyr phosphatase (B) activity was measured in the cell lysate. *p≤0.001 vs untreated control, #p≤0.001 vs sPLA_2_ alone, n = 3.

Since the homeostasis of protein phosphorylation events is governed by kinases and phosphatases, we looked for changes in the phosphatase activity, as a putative mechanism involved in the changes of the temporal phosphorylation pattern orchestrated by the presence of leptin. As shown in Fig 8, we found that leptin stimulation, both alone and in the simultaneous presence of sPLA_2_-IIA, decreased serine/threonine and tyrosine phosphatase activity in 1321N1 cells. In contrast, incubation of intact cells with sPLA_2_-IIA alone did not affect the phosphatase activity compared to non-stimulated cells. These findings suggest that sustained phosphorylation levels in MAPKs, Akt and EGFR promoted by co-stimulation of leptin and sPLA_2_-IIA in 1321N1 astrocytoma cells might be partly attributed to a reduced phosphatase activity.

### Synergistic stimulatory effects of the combination of leptin and sPLA_2_-IIA on primary astrocyte cultures

**Fig 9:**
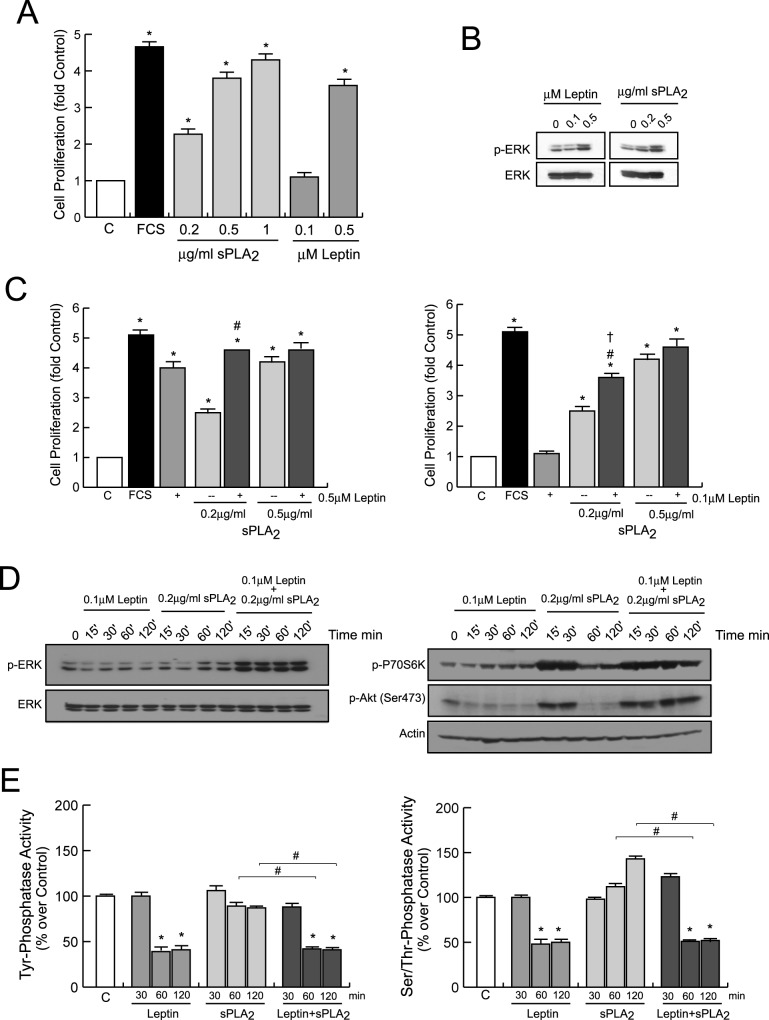
Actions of leptin and sPLA_2_-IIA on mouse primary astrocytes. Astrocytes were stimulated with leptin, sPLA_2_-IIA (sPLA_2_) or both, as indicated. (A, C) After 24 h cell proliferation was measured as explained in Materials and Methods. *p≤0.001 vs untreated control cells, #p≤0.001 vs 0.2 μg/ml of sPLA_2_ alone, ^†^p≤0.001 vs 0.1 μM of leptin alone, n = 3. (B, D) After 15 min, phosphorylation of signaling proteins was determined by immunoblotting on the cell extracts. (E) At the indicated times, the activity of Ser/Thr- and Tyr-phosphatase was measured in the cell lysate. *p≤0.001 vs untreated control, #p≤0.001 vs sPLA_2_ alone, n = 3.

Next, we extended these observations related to the synergistic interactions between leptin and sPLA_2_-IIA to primary cultures of astrocytes. As shown in Fig 9A, cell proliferation was induced in serum-starved astrocytes upon treatment with leptin and sPLA_2_-IIA. Alike in 1321N1 astrocytoma cells, a synergistic effect on cell growth was found when astrocytes were stimulated with a suboptimal dose of leptin in combination with a suboptimal dose of sPLA_2_-IIA (Fig 9C). Then, we analyzed the activation/phosphorylation of some representative intracellular signaling mediators associated with proliferation. Leptin and sPLA_2_-IIA increased ERK phosphorylation in a dose-dependent manner in primary cultures of astrocytes (Fig 9B). The simultaneous presence of both agonists at suboptimal doses also led to an immediate and sustained increase in the phosphorylation of the studied signaling proteins (Fig 9D, S5 Fig), as previously reported in 1321N1 astrocytoma cells. As expected, the presence of leptin, both alone or in the presence of sPLA_2_-IIA, dramatically diminished the activity of both types of phosphatases upon 30 min treatment (Fig 9E).

## Discussion

This study provides possible mechanisms to better understand how obesity-related inflammation may affect risk and prognosis of multiple diseases. We focused on cancer and glial tumors in particular, since a recent study shows that obesity worsens the prognosis of glioma patients [4]. To date, the pathophysiology and molecular mechanisms underlying these associations are not fully understood, although several hormonal systems—have been studied to some extent, and a leptin-cytokine cross talk has been observed in breast cancer [26] The role of leptin and sPLA_2_-IIA in the initiation and metastasis of tumors has been documented and reviewed, and here we pay attention to both proteins as emerging candidate molecules linking obesity and inflammation in glioma tumors [6,8,21].

This study illustrates key signaling molecules involved in leptin-induced proliferation and migration of human astrocytoma cells, and demonstrates a cross-talk between leptin and the inflammatory sPLA_2_-IIA. We observed that leptin increases the proliferative and migratory effects of sPLA_2_-IIA on both astrocytoma cells and primary astrocytes. The synergy between them is found at doses that individually show no activation, or activation below the reported maximal effects. Synergy between inflammation-related factors has already been pointed as an additional mechanism of the inflammatory system to sustain or amplify inflammatory responses when suboptimal cytokine/chemokine concentrations are present [27–29].

Effects of leptin on different glioblastoma cell lines had been previously examined in vitro, and leptin has been found: i) to induce migration and invasion of C6 rat cells linked to the activation of p38MAP kinase and NF-κB pathway, ii) to promote human U87 cells proliferation mainly through Janus-Activated Kinase (JAK)/Signal Transducer and Activator of Transcription 3 (STAT3) signaling pathway and iii) to enhance growth and migration of human LN18 and LN229 cells along with the activation of the PI-3K/Akt and STAT3 pathways [30–32]. The present study reveals that leptin-induced human 1321N1 astrocytoma cells growth and mobility requires the activation of key intracellular signal transduction elements from the major signalling cascades: MEK/ERK and PI3K/Akt/mTOR/P70S6K pathways, but not PKC activation as required by other proliferative molecules, such as EGF or sPLA_2_-IIA itself [24] Our data also suggest that both cascades converge to regulate pro-tumoral events, since inhibition of any of those pathways significantly diminishes leptin effects. Interestingly, although in some cell types leptin signaling is dependent on EGFR transactivation, in 1321N1 astrocytoma cells the presence of the specific EGFR inhibitor, AG1478, does not abrogate the mitogenic effects induced by leptin alone [33].

The directed chemotactic migration of tumoral cells is an essential component of tumor dissemination during progression and invasion. Here, we show that leptin not only behaves as a chemoattractant for 1321N1 cells, but it also improves FCS-induced chemotaxis. These results are inline with previous studies demonstrating cooperative chemotactic responses between leptin and some mediators from the molecular network by which obesity impacts/modifies? the pathological manifestation of carcinogenesis, such as IGF-I [19]. In addition, we found that leptin-promoted chemotaxis of 1321N1 cells is dependent on activation/phosphorylation of similar crucial kinases, hence signaling cascades, to leptin-induced mitogenesis.

Our study also found out that surface leptin receptor is enhanced by pro-inflammatory sPLA_2_-IIA and growth factors, such as EGF and PDGF. Since inflammation and over-expression of growth factors in glioma tumors involves pro-inflammatory proteins production, including sPLA_2_-IIA; and these factors induce upregulation of the leptin receptor, it is conceivable that leptin has a cooperative role. Leptin enhances sPLA_2_-IIA-mediated effects in 1321N1 astrocytoma cells, and sPLA_2_-IIA enhances leptin capacity to signal by inducing overexpression of its receptor, thereby possibly contributing to a more aggressive behaviour of the basal tumor phenotype.

sPLA_2_-IIA has been studied in different types of human malignancies, and it has been shown to exert diverse, and even contradictory, biological effects, probably due to tissue- or cell-specific functions [20,23,34]. We have previously reported that sPLA_2_-IIA mediates 1321N1 cell proliferation, and here we demonstrate that it also modulates astrocytoma cell mobility. Additionally, we found that the rapid and transient activation of crucial kinases—such as Src, ERK, Akt, P70S6K - induced by sPLA_2_-IIA are prolonged in time in the simultaneous presence of leptin. These amplifying effects of leptin in the phosphorylation state of those signaling elements might be the cause of its synergistic effect, since a long-lasting phosphorylation could enhance the activity of the intracellular pathways involved in the proliferative and migratory responses to the phospholipase in 1321N1 cells.

Synergistic interactions between leptin and several factors have been previously described in the regulation of diverse cell functions. Leptin interacts with cholecystokinin at the level of vagal afferent neurons to control the function of the early growth response protein-1 (EGR1), and synergistically increase phosphorylation of STAT3 to enhance neural excitability on the nodose ganglia neurons [35,36]. Leptin also synergizes with stem cell factor in the stimulation of murine hematopoietic progenitor cells proliferation, and with thyroid hormone to promote growth plate chondrocyte proliferation and terminal differentiation [37,38]. A similar synergism on the activation of intracellular kinases has been reported between leptin/IL-1 and leptin/IFNγ to activate NOS type II in chondrocytes, and a synergistic activation of ERK and EGFR has been associated with cell growth in oesophageal adenocarcinoma cells exposed to leptin together with acid [39–41].

In 1321N1 cells, we identified EGFR phosphorylation upon sPLA_2_-IIA stimulation, and we observed that the simultaneous presence of leptin prolonged its phosphorylation time-course, although a direct action of leptin on EGFR phosphorylation was excluded analyzing results from cells treated with leptin alone. Activation of the EGFR, by extracellular stimuli in addition to EGF-like ligands, is widely recognized as a potent signal in the regulation of diverse biological processes including cell proliferation, survival and motility, depending on the cellular system, the stimulus and the repertoire of signaling molecules recruited [42]. Therefore, it could be hypothesized that the sustained phosphorylation promoted by leptin on sPLA_2_-IIA-EGFR transactivation is an important step in the cooperative effects of both proteins.

This prolonged phosphorylated status promoted by leptin when sPLA_2_-IIA is present, would also be consistent with leptin acting through the regulation of the activity of serine/threonine phosphatases and/or tyrosine phosphatases. It has been demonstrated that leptin reduces the enzymatic activity of the serine/threonine phosphatase PP-1 in INS-1 cells, and inhibits the phosphatase activity of the tyrosine phosphatase PTEN in mouse hypothalamic cells and pancreatic β-cells [43,44]. Furthermore, it has been reported that inhibition of the dual phosphatase MKP-1 increases ERK phosphorylation, and a prolonged EGFR phosphorylation is triggered upon inhibition of the dual protein phosphatase cdc25A, and the tyrosine phosphatases PTP1B and SHP-2 [45–48]. In this study we find that in the presence leptin, the activity of protein phosphatases is notably diminished. However, further studies should be performed to elucidate whether leptin is either indirectly regulating protein phosphatase inhibitors, or directly affecting the activity of protein phosphatases, and, if so, to identify the phosphatase(s) responsible involved.

This synergic interaction between leptin and sPLA_2_-IIA was also observed in primary astrocytes. Since sPLA_2_-IIA has been found upregulated in diseases that affect the nervous system, such as stroke and Alzheimer's disease, and these disorders are considered obesity-related diseases, our results might also have relevance and contribute to explain some of the obesity-related medical complications also in non-tumoral pathologies of the nervous system [49–51].

In conclusion, the synergic interaction between obesity- and inflammatory-related mediators provides an additional mechanism to sustain a harmful response in the presence of suboptimal concentrations of either molecule. As a consequence, low concentrations of inflammatory mediators in the tumor microenvironment, or in damaged brain areas, could be stimulating cell growth and dissemination in overweight or obese subjects, thus being a mechanism by which ***obesity*** accelerates development and malignancy of ***cancer*** and other *CNS* ***pathologies***.

## Supporting information

S1 Fig Estatistical analysis of Western Blots from Fig 2. Quantification of Western Blots in Panel A (A), and in Panel D (B). Bars are the the mean ± SD in arbitraty units, n = 3. *p≤0.001 vs control without inhibitor. +p≤0.001 vs EGF.
(EPS)

S2 Fig Estatistical analysis of Western Blots from Fig 3. (A, B, C and D) Quantification of Western Blots in Panels A, B, C and D, respectively. Bars are the mean ± SD in arbitraty units, n = 3. *p≤0.001 vs. control without inhibitor. †p≤0.001 and ††p≤0.05 vs leptin alone. +p≤0.001 vs EGF alone.
(EPS)

S3 Fig Estatistical analysis of Western Blots from Fig 6. Quantification of Western Blots in Panel B. Bars are the mean ± SD in arbitraty units, n = 3. #p≤0.001 vs sPLA_2_ alone.
(EPS)

S4 Fig Estatistical analysis of Western Blots from Fig 7. Quantification of Western Blots in Panel C. Bars are the the mean ± SD in arbitraty units, n = 3. ‡‡p≤0.01 and ‡p≤0.001 vs leptin+sPLA_2_-IIA without inhibitor.
(EPS)

S5 Fig Estatistical analysis of Western Blots from Fig 9. Quantification of Western Blots in Panel D. Bars are the the mean ± SD in arbitraty units, n = 3. #p≤0.001 vs sPLA_2_ alone.
(EPS)
